# Antibacterial, Antibiofilm, and Wound Healing Activities of Rutin and Quercetin and Their Interaction with Gentamicin on Excision Wounds in Diabetic Mice

**DOI:** 10.3390/biology13090676

**Published:** 2024-08-29

**Authors:** Yasir Almuhanna, Abdulrahman Alshalani, Hamood AlSudais, Fuad Alanazi, Mohammed Alissa, Mohammed Asad, Babu Joseph

**Affiliations:** 1 Department of Clinical Laboratory Sciences, College of Applied Medical Sciences, Shaqra University, Shaqra 11961, Saudi Arabia; masad@su.edu.sa; 2 Chair of Medical and Molecular Genetics Research, Department of Clinical Laboratory Sciences, College of Applied Medical Sciences, King Saud University, Riyadh 12372, Saudi Arabia; aalshalani@ksu.edu.sa (A.A.); halsudais@ksu.edu.sa (H.A.); 3 Department of Clinical Laboratory Sciences, College of Applied Medical Sciences, King Saud University, Riyadh 12372, Saudi Arabia; foalanazi@ksu.edu.sa; 4 Department of Medical Laboratory, College of Applied Medical Sciences, Prince Sattam bin Abdulaziz University, Al-Kharj 11942, Saudi Arabia; m.alissa@psau.edu.sa

**Keywords:** antibacterial compounds, antibiotic resistance, bioactive compounds, histology, quercetin, rutin, wound healing

## Abstract

**Simple Summary:**

Phytochemicals are used against drug-resistant bacterial strains. In this work, diabetic mice with excision wounds infected with multidrug-resistant (MDR)-*P. aeruginosa* were used. The antibacterial, antibiofilm, and wound-healing properties of rutin and quercetin were studied. The broth dilution and crystal violet assay were used to investigate the antibacterial and antibiofilm activities in vitro, respectively. At higher concentrations, quercetin and rutin showed promise against MDR-*P. aeruginosa*. By inhibiting biofilm from the injured tissue, both phytochemicals aided in the healing of diabetic wounds by preventing the formation of biofilm in vitro. Compared to rutin, quercetin had an impact on accelerating wound healing and promoting epithelial layer regeneration. When combined with gentamicin, both phytochemicals were found to be more successful in regulating biofilm and wound-healing processes. The research validates the conventional application of phytochemicals possessing antibacterial, antibiofilm, and wound-healing properties for the treatment of diabetic infections.

**Abstract:**

Phytochemicals are effective and are gaining attention in fighting against drug-resistant bacterial strains. In the present study, rutin and quercetin were tested for antibacterial, antibiofilm, and wound healing activities on excision wounds infected with MDR-*P. aeruginosa* in diabetic mice. Antibacterial and antibiofilm activities were studied in vitro using broth dilution assay and crystal violet assay, respectively. These phytochemicals were tested alone for wound-healing activities at different concentrations (0.5% and 1% in ointment base) and in combination with gentamicin to evaluate any additive effects. Rutin and quercetin demonstrated effectiveness against MDR-*P. aeruginosa* at higher concentrations. Both phytochemicals inhibited biofilm formation in vitro and contributed to the healing of diabetic wounds by eradicating biofilm in the wounded tissue. Rutin at a low concentration (0.5%) had a lesser effect on reducing the epithelization period and regeneration of the epithelial layer compared to quercetin. When combined with gentamicin, quercetin (1%) displayed the maximum effect on epithelium regeneration, followed by rutin (1%) in combination with gentamicin. Both phytochemicals were found to be more effective in controlling biofilm and wound-healing activities when used as an additive with gentamicin. The study supports the traditional use of phytochemicals with antibacterial, antibiofilm, and wound-healing activities in managing diabetic infections.

## 1. Introduction

Diabetes is a prevalent and complex metabolic disorder, and impaired wound healing and the formation of biofilm by pathogenic bacteria are significant issues in diabetic patients. Wound healing is a complex and prolonged process, particularly challenging in diabetic conditions due to vascular complications and other factors [1]. The fundamental responses in wound healing involve the repair of connective tissues through hemostasis, inflammation, proliferation, and remodeling coordinated by various cells, growth factors, and cytokines [2]. Wounds infected by antibiotic-resistant bacterial pathogens form biofilm, exacerbating impairment of skin and reducing antibiotic effectiveness, leading to treatment failure, infection recurrence, and a heightened risk of limb amputation [3]. The increase in drug resistance, adaptation, and development of survival mechanisms by pathogens is increasing the complexity and expense of treating infections. Clinical management of such infections with broad-spectrum antibiotics is challenging, and prolonged usage leads to serious therapeutic problems [4].

Phytochemicals are recognized for their antimicrobial and antibiofilm activities, and for accelerating wound healing [5,6]. Consequently, various plant metabolites and novel bioactive components are gaining attention to address infection control and prevent the development of resistance to antibiotics [7]. Metabolites such as alkaloids, coumarins, cardiac glycosides, flavonoids, phenols, terpenoids, saponins, sterols, and tannins have demonstrated antibacterial activity against Gram-positive and Gram-negative bacteria [8,9]. The phytoconstituents are considered to be safer compared to other wound healing agents [10].

Rutin is a polyphenol flavonoid that is well known for its antioxidant activity and anti-inflammatory action [11,12]. During diabetes, the combined impact of oxidative damage, inflammation, and high blood sugar significantly impedes wound healing [13]. Hence, the antioxidant activity and anti-inflammatory properties of rutin may help in the healing of diabetic wounds. Further, the antibacterial activity of rutin is reported against both Gram-positive and Gram-negative bacteria [14], which may prevent infection to enhance wound healing. Quercetin is another polyphenol flavonoid that has been reported for several biological effects. Some of its reported activities include anti-allergic, anti-inflammatory, anticancer, and antimicrobial properties [15]. It is also reported to protect against cardiovascular diseases and neurological damage [16]. Quercetin is reported to have good in vitro antimicrobial, anti-inflammatory, and antioxidant effects [17]. All these effects are known to positively influence the healing of infectious wounded tissue. However, there are few reports on the wound-healing effects of quercetin in multidrug-resistant infected wounds in animals [18,19]. It is well known that phytochemicals provide a promising avenue in the fight against antibiotic resistance through their direct antimicrobial properties, their capability to enhance the activity of conventional antibiotics, and their modulation of bacterial virulence factors [20]. Hence, a detailed study about the potential of phytochemicals possessing antimicrobial and wound healing activities on multidrug-resistant pathogen-infected diabetic wounds may lead to the identification of novel drug molecules. Multidrug-resistant (MDR) *Pseudomonas aeruginosa* is a Gram-negative bacterium, and it is a well-established fact that many antibacterial agents are more effective against Gram-positive bacteria than Gram-negative bacteria due to their cell wall structure. This is primarily attributed to the presence of the lipopolysaccharide layer in the cell wall and the periplasmic space, rendering Gram-negative bacteria more resilient to antimicrobial agents compared to Gram-positive bacteria [21].

Therefore, this study explored the antibacterial and antibiofilm activities of polyphenols, rutin, and quercetin against MDR-*P. aeruginosa* causing wound infections. The infection was induced in excision wounds in diabetic mice and wound healing activity was studied after biofilm formation. Additionally, the interaction of these phytochemicals with gentamicin was explored.

## 2. Materials and Methods

### 2.1. Chemicals and Bacterial Cultures

All chemicals used in the study are of analytical grade. Rutin and quercetin were purchased from MedChemExpress (# HY-14590, Monmouth Junction, NJ, USA). Rutin and quercetin were suspended in sterile 0.1% sodium carboxymethylcellulose for in vitro antimicrobial studies. The flavonoids were formulated into an ointment by mixing with glycol stearate, 1,2-propanediol (propylene glycol), and paraffin oil at a 3:6:1 ratio. MDR-*P. aeruginosa* (ATCC 27853) available in the Department of Clinical Laboratory Science, Shaqra University was used. The bacteria were grown in Luria-Bertani (LB) broth at 37 °C and the cell culture was adjusted to 10^8^ CFU/mL.

### 2.2. Animals

Swiss albino mice (7–8 weeks old; weighing around 20 g) of either sex were used. All experimental protocols were carried out as per ARRIVE guidelines [22] after receiving approval for the ethical and scientific content from the Research Ethics Committee of Shaqra University (ERC-SU_F_202300003).

### 2.3. Minimum Inhibitory Concentration (In Vitro)

The antibacterial activity of the rutin and quercetin was evaluated by using the broth dilution method [23]. Both minimum inhibitory concentration (MIC) and minimum bactericidal concentration (MBC) were determined against MDR-*Pseudomonas aeruginosa*. The fractional inhibitory concentration (FIC) index was determined by checkerboard assay [24].

### 2.4. Antibiofilm Activity (In Vitro)

The bacterial strains from the overnight cultures were transferred to Luria Bertani broth and kept in a shaking incubator (200 rpm at 37 °C) for 3 h. The turbidity (OD 600 nm) was adjusted to 0.04 and 100 µL of the culture was transferred into microtitre plate wells along with rutin and quercetin at sub-MIC levels and incubated (37 °C). After 24 h incubation, the culture was discarded and the wells were washed three times with sterile phosphate buffer without disturbing the biofilm. The wells are then filled with 125 µL of 1% w/v crystal violet (1 h) for staining the biofilm. Following this, the stain was removed using a micropipette, and the wells were carefully washed using phosphate buffer to remove excess stain. The dye adhered to the biofilm was dissolved in 70% ethanol and, after 15 min, transferred to fresh wells. The absorbance was measured at 570 nm using an ELISA plate reader [25].

### 2.5. Formulation of Rutin and Quercetin Ointment

The ointment base was formulated by mixing glycol stearate, 1,2-propanediol, and paraffin oil at a 3:6:1 ratio. Rutin and quercetin ointments (0.5% and 1% w/w) were prepared by adding these phytochemicals to the base preparation. The ointment base was chosen based on previous reports [26]. The prepared ointment was tested for its physicochemical properties—color, homogeneity, washability, and spreadability—following established protocols [27]. To evaluate washability, a precise amount of the ointment was applied to a researcher’s hand and subsequently washed with water. The spreadability was quantified in seconds, measuring the time it took for slides to slip from a dollop of ointment placed between them in line with the applied load. The extent of spreadability was determined using the formula S = M × L/T, with S signifying spreadability, M representing the load with the upper slide, L indicating the length of the glass slides, and T denoting the time taken to separate the slides. The diffusion ability was tested in an agar medium [28], and stability was assessed at various temperatures over three months [29]. A skin irritation study was carried out using standard protocol [30]. The gentamicin ointment (0.1%) was prepared following a method described earlier [31].

### 2.6. Induction of Diabetes

Streptozocin and nicotinamide were used to induce diabetes (type II) in mice [32]. The mice underwent a 12 h fasting, followed by intraperitoneal injections of nicotinamide (240 mg/kg) and streptozocin (100 mg/kg). Citrate buffer was used as a vehicle to prepare the injections (1 mL/100 g) [33]. After 3 days, blood glucose levels were measured following another 12 h fast. Animals showing blood sugar levels of 150 mg/dL or higher were carefully chosen to assess antibiofilm activity. Throughout the experiment, the animals were fed with water and fed normally (*ad libitum*). Strict procedures were taken to prevent infection transmission. The animals were diligently monitored for any signs of mortality.

### 2.7. Antibiofilm Activity In Vivo

Antibiofilm activity on excision wounds was carried out using a previously described method [34]. A preformed biofilm was developed on a coverslip immersed in LB broth inside a culture bottle inoculated with MDR-*P. aeruginosa*. The presence of biofilm was confirmed through the use of two distinct methods, namely Gram staining and the crystal violet assay [35]. Initially, a coverslip was incubated in LB broth, then subjected to Gram’s reagent staining, and observed under a microscope for the presence of aggregate biofilm formation. In parallel, another coverslip was carefully washed with phosphate buffer saline and immersed in a 1% w/v crystal violet solution for 15 min. Upon staining, any excess crystal violet was removed by washing with phosphate buffer saline, and the coverslips were subsequently immersed in 70% ethanol to dissolve the crystal violet. The intensity of the crystal violet color was measured using a spectrophotometer at 570 nm and compared with a control coverslip devoid of biofilm. Animals were anesthetized to induce excision wounds on their backs by depilating the skin [36]. Briefly, an area (1 cm^2^) in the dorsal region of the mouse was subjected to depilation utilizing a hair-removing cream (Veet, Reckitt Benckiser Group PLC, Jeddah, Saudi Arabia). Subsequently, the depilated area was cleansed with normal saline and the mice were subjected to a 12 h observation period to detect any indications of irritation or inflammation associated with the hair removal cream. The following day, the mice were anesthetized using a combination of ketamine (87.5 mg) and xylazine (12.5 mg) in saline via intraperitoneal administration at a dosage of 0.1 mL/20 g. The depilated skin area was carefully excised to full thickness. Following this, the preformed biofilm on the coverslip was placed over the excision wound surface and 10 µL of MDR-*P. aeruginosa* inoculum was inoculated on the wound (100 µL with 10^6^ CFU/mL). After 72 h, the wounds were observed macroscopically for biofilm formation and confirmed by pus and exudate formation in at least 10% of the animals selected for the study. A thin layer formed on the wound was removed using forceps and examined by Gram staining to confirm the presence of bacteria. Subsequently, the animals were divided into eight different groups containing at least twelve animals in each group until the experiment was completed. The treatments for different groups included the following: group I received the ointment base, group II received gentamicin ointment (0.1%), and groups III and IV were given an application of rutin at 0.5% or 1%, respectively. Groups V and VI were applied with quercetin at 0.5% or 1%, respectively. The last two groups (VII and VIII) were treated with a combination of gentamicin (0.1%) with either rutin (1%) or quercetin (1%). The wound contraction (%) was calculated [37] by measuring the area at 4-day intervals. A transparent sheet was used to trace the wound area and superimposed on a graph sheet to calculate the wound area [38]. The animals were sacrificed on the 20th day and the bacterial count on the wounded skin (CFU/g) was determined. One gram of the tissue was homogenized in sterile phosphate buffer saline using a tissue homogenizer under sterile conditions and serially diluted. The dilutions were plated on cetrimide agar and incubated for 24 h at 37 °C [39]. The tissues from the newly formed skin were also subjected to histological study (H&E staining). The regenerated epithelial thickness was qualitatively observed (Leica microscope DM 2500 LED with camera-DFC 295 using Leica LAS EZ software; version 3.0.0). The regenerated epithelial thickness in the control animals treated with base was compared to other treatment groups. The epithelization period was determined as the day on which there was a falling of the scar, leaving no raw wound [40]. The absence of exudates or pus and hair growth on the edges of the wound indicated re-epithelization.

Statistical analysis: All results show mean ± SEM in the footnotes. One-way ANOVA with Tukey’s post-test was completed using SPSS (version 20 for Windows).

## 3. Results

### 3.1. Antibacterial Activity

The results of the study showed that rutin exhibited antibacterial effects against MDR-*P. aeruginosa* (MIC-512 µg/mL) while quercetin showed stronger antibacterial effects than rutin, with a MIC at 256 µg/mL. The minimum bactericidal concentration (MBC) for rutin was 1024 µg/mL, while the MBC for quercetin was 512 µg/mL (Table 1). There was no synergetic interaction between rutin or quercetin with gentamicin in the present study in the checkerboard assay.

### 3.2. Antibiofilm Activity

**Figure 1:**
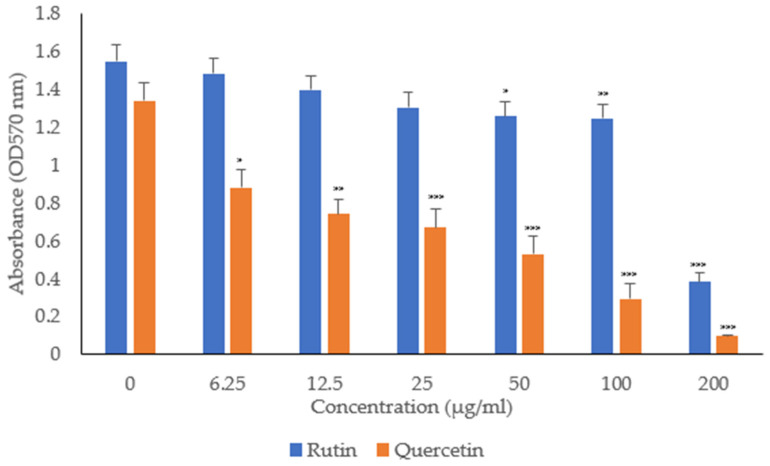
In vitro antibiofilm activity of rutin and quercetin against MDR-*P. aeruginosa*. Each experiment was repeated three times (*n* = 3) and each sample was in duplicate. Bars indicate means ± SEM, * *p* < 0.05, ** *p* < 0.01, *** *p* < 0.001 as compared to control (0 µg/mL). Statistical analysis was completed using one-way ANOVA followed by Tukey’s test.

The antibiofilm activity of rutin and quercetin was evaluated at sub-MIC concentration against both bacterial pathogens. Rutin displayed significant antibiofilm efficacy against MDR-*P. aeruginosa* above 50 µg/mL. However, quercetin was more potent and effectively inhibited the biofilm formation of MDR-*P. aeruginosa* at all the tested concentrations (Figure 1). The antibiofilm activity of both the phytochemicals was observed to be dose-dependent.

### 3.3. Epithelization Period

The ointment preparation was stable and had good diffusing and spreading abilities. It was homogenous and had excellent washability (Table 2).

**Figure 2:**
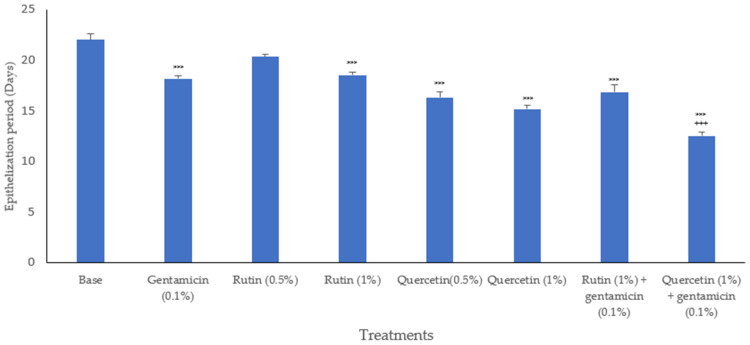
Epithelization period in biofilm-induced excision wound in mice after different treatments. The epithelization period is the day on which there was a falling of the scar, leaving no raw wound. Bars represent mean ± SEM, *n* = 6, *** *p* < 0.001 as compared to base-treated control. +++ *p* < 0.001 as compared to individual quercetin (1%) treatment. Statistical analysis was completed using one-way ANOVA followed by Tukey’s test.

When locally applied, the quercetin ointment at both doses showed significant wound contraction in pathogen-infected wounds compared with the base-treated control. However, rutin had a lesser effect, and the 0.5% dose of rutin did not show any significant effect on the epithelization period. In contrast, quercetin at both doses of 0.5% and 1% had a significant effect in reducing the epithelization period. When rutin at both doses of 0.5% and 1% was combined with gentamicin (0.1%), no significant change in the combination group was observed in comparison to rutin treatment alone. However, a combination of quercetin (1%) with gentamicin (0.1%) produced a significant decrease in the epithelization period (Figure 2).

### 3.4. Wound Healing

**Figure 3:**
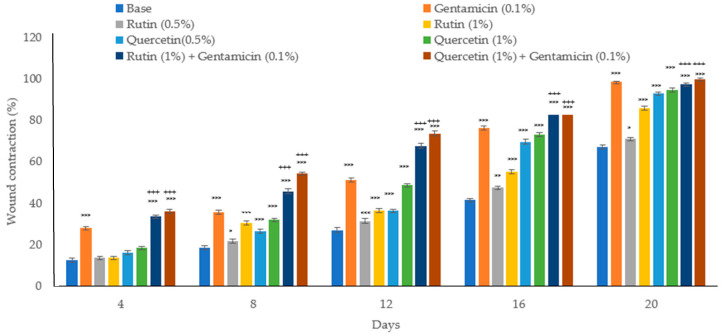
Effect on wound contraction. MDR-*P. aeruginosa*-infected wounded tissue contracted differently after various treatments. The bars in the graph represent mean ± SEM, *n* = 6. * *p* < 0.05, ** *p* < 0.01, *** *p* < 0.001 indicate significance compared to the base-treated control. +++ *p* < 0.001 indicate significance compared to the individual quercetin (1%) or rutin (1%) treatment. Statistical analysis was completed using one-way ANOVA followed by Tukey’s test.

The rutin and quercetin ointments enhanced wound healing in infected wounds of diabetic mice related to the control group. However, the effect was relatively less. Both concentrations of these phytochemicals significantly helped in wound contraction starting from the 8th day (*p* < 0.001) compared to the control group. Wound contraction is the decrease in wound area on different days of drug treatment. The combination of rutin and quercetin with gentamicin was also more effective than either phytochemical alone (Figure 3).

**Figure 4:**
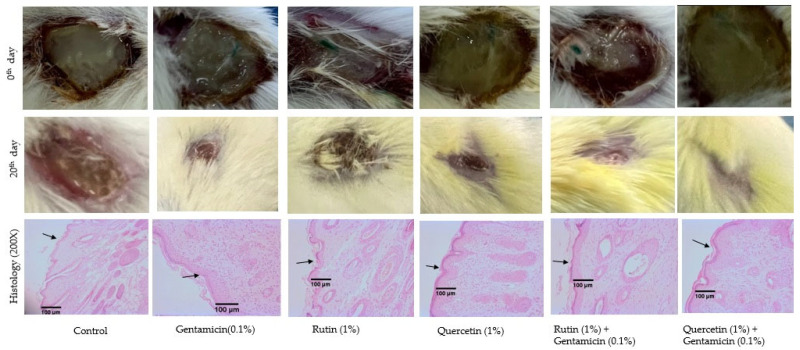
Images showing macroscopic appearance and microscopic changes in the wounded tissues. The wounded tissue images on Day 0 show pus formation in all the groups. Treatment with gentamicin (0.1%), rutin (1%), quercetin (1%), and their combinations accelerated the healing of wounds to varying degrees. Wounded tissue histology at 200× from different treatment groups is shown. The regenerated epithelial tissue is shown by black arrows. The regenerated epithelial tissue height was lowest in the infected control groups. Gentamicin (0.1%) and the combination of gentamicin (0.1%) with quercetin (1%) increased the regeneration of skin epithelium as indicated by the thickness of the tissue in histological sections. Rutin (1%) produced less regenerative effect compared to other treatments. Note: the images are from groups that showed maximum treatment effect, and the epithelial tissue histology from other groups with intermediate effects has not been shown.The bacteria count in the wounded tissue 20 days after using the phytochemical ointment was significantly less compared to the control treated with the base. However, there was no significant reduction in the count of *P. aeruginosa* at the 0.5% concentration of rutin, while the higher concentration (1%) of rutin and both the concentration of quercetin (0.5% and 1%) significantly decreased the bacterial count. The growth and counting of other bacteria was avoided by using cetrimide agar which is selective for *P. aeruginosa*. Treatment with gentamicin significantly decreased the bacterial load compared to the control. A combination of quercetin (1%) + gentamicin (0.1%) was significantly more effective in reducing CFU in the tissue when compared to quercetin (1%) alone while no such action could be observed with the combination of rutin (1%) and gentamicin (0.1%) as compared to rutin (1%) alone (Table 3).

Histological examination of the wounded tissue supported macroscopic findings on the wound closure. Rutin at both doses produced less regeneration of skin epithelium while the combination of quercetin (1%) with gentamicin (0.1%) showed the maximum regeneration of skin epithelial tissue as compared to the control. Only a qualitative observation was made to determine the changes in the skin epithelial tissues. The treatment with the combination of quercetin (1%) with gentamicin (0.1%) healed the wounded tissue completely and the regenerated epithelium resembled normal skin epithelium. Macroscopic observations of the wounded tissue supported the microscopic changes in the wounded tissues (Figure 4).

## 4. Discussion

Wound healing is a complex process that involves the immune system and antimicrobial therapy. The rise of drug-resistant bacteria in chronic wounds has led to the development of phytomedicine as a potential treatment [41]. Many medicinal plants have been used to treat wounds, help in the wound-healing process, and fight infections. With the rise of multidrug-resistant bacteria, there is increased interest in traditionally used medicine for the management of diabetic wounds. Previous reports show that a significant percentage of the population in Saudi Arabia depends on traditional medicine or uses it in conjunction with modern medicine to address infections [42]. Phytochemical components are well known for their biological activities. In a previous investigation, significant antibiofilm and wound-healing effects were observed from different plant extracts containing polyphenols when used on wounds infected with methicillin-resistant *Staphylococcus aureus* (MRSA) and *P. aeruginosa* in normal and diabetic mice. However, the specific chemical compound(s) responsible for these effects remains unknown, even though the presence of polyphenols is often associated with the pharmacological activities of plant extracts. The present study showed the antibacterial and antibiofilm effects of rutin and quercetin glucoside on multidrug-resistant bacteria, as well as their wound-healing properties in diabetic mice models. Additionally, histological changes supported their potential role in diabetic wound healing. There are earlier studies on the effects of rutin and quercetin on human keratinocytes [43,44]. Hence, these assays were not performed in the current study. These flavonoids were shown to possess cytoprotective effects on human keratinocytes. Different ointment bases were used for the preparation of flavonoids and gentamicin. Ointment bases are considered inert and have similar effects. An earlier study with three different ointment bases showed that there was no significant difference between the ointment bases [45]. Hence, the control group was treated with ointment that was used for the preparation of flavonoids. Furthermore, ethical issues with the use of more animals were a barrier to having more control groups.

Rutin and quercetin are flavonoids with antibacterial, antioxidant, and anti-inflammatory properties that may aid in the healing of wounds. Furthermore, these two flavonoids are polyphenols known for several biological effects, especially modulation of inflammatory reactions and wound healing [46,47].

Rutin is a flavonoid found in several plants, especially in citrus fruits. Earlier studies on its antibacterial effects show that rutin has an antibacterial effect against *Aeromonas hydrophila* and it also potentiates the effect of another flavonoid florfenicol [48]. Rutin is also known to promote wound healing through its antibacterial and anti-inflammatory properties. It has shown promising results in accelerating wound healing and reducing inflammation in different conditions [49]. It also enhanced the antibacterial effect of amikacin against different strains of bacteria [14]. Another study on rats showed that rutin improves metabolic dysfunctions in diabetic rats, promotes wound healing, and attenuates inflammation and oxidative stress [50]. Furthermore, a polymerizing rutin called polyrutin has better antioxidant and free radical scavenging potential, and is believed to be one of the potential wound healing agents. The current study demonstrated that rutin has antibacterial and antibiofilm properties against MDR-*Pseudomonas aeruginosa*. In the in vitro study, rutin inhibited biofilm formation at higher concentrations of 50 µg/mL onwards and its MIC was also higher at 512 µg/mL. The application of 1% rutin accelerated the wound healing process. This result suggests that other effects of rutin mentioned above, such as antioxidant and anti-inflammatory effects, might have supported wound healing. Several reports are available on the antioxidant activity of these components [46] and it was not evaluated in the present study. Furthermore, the weak antibacterial and anti-biofilm activity observed in the current study further confirms that rutin may not be a very effective antibacterial drug. This is also supported by the very few reports on the antibacterial effects of rutin. Combining rutin with the common antibiotic gentamicin (0.1%) enhanced wound healing and reduced microbial load more effectively than rutin alone demonstrating an additive effect. The enhanced wound healing could be due to a combination of the anti-inflammatory, antioxidant, and mild antibacterial actions of rutin, along with the strong antibacterial effect of gentamicin against MDR-*P. aeruginosa*.

Quercetin has various beneficial properties, including anti-inflammatory, antibacterial, angiogenesis, regulating immune response, and free radical scavenging effects [51]. When applied topically, it is reported to raise the level of cytokines, interleukin-10 (IL-10) and suppress the expression of tumor necrosis factor-α [51]. Furthermore, quercetin increases the levels of vascular endothelial growth factor (VEGF), transforming growth factor-β (TGF-β), and antioxidants, and attenuates the expression of inflammatory factors [52]. In a recent study in mice, quercetin promoted fibroblast proliferation and migration, and these effects are known to support wound healing. It also restored normal dermal structure and enhanced collagen fiber content. Quercetin decreased inflammatory mediators such as tumor necrosis factor-α, interleukin-1β, and interleukin-6. It also exhibited a potent antioxidant effect. Quercetin was also shown to increase the protein levels of Wnt and β-catenin, confirming its potential as a wound-healing agent in mice [53].

Rutin is a glycoside of quercetin [54]. This study demonstrated that quercetin is a more effective wound-healing agent than rutin. Earlier studies on the comparative antimicrobial, antioxidant, and anti-inflammatory effects of rutin and quercetin showed similar results [46,47]. The reason for the difference in the activities of quercetin and a glycoside of quercetin–rutin cannot be explained with the present data. However, it is speculated that enhanced antimicrobial, antioxidant, and anti-inflammatory actions of quercetin as compared to rutin may have contributed to the difference in the effects observed.

Quercetin has been reported for antibacterial effects against several bacterial strains [55,56]. The antibacterial action has been attributed to its interaction with the bacterial cell membrane, and earlier reports show that Gram-negative bacteria are more resistant to the bactericidal effect of quercetin [55]. In the current study, the MIC was found to be 256 µg/mL and MBC was at 512 µg/mL. The application of quercetin with gentamicin showed a greater wound healing effect compared to either applied alone, suggesting an additive effect. This additive effect could be due to different mechanisms of antibacterial action. As mentioned above, quercetin increases cell membrane permeability in bacterial cells [55], while gentamicin acts through the inhibition of bacterial protein synthesis [57].

## 5. Conclusions

The polyphenols rutin and quercetin used in this study demonstrated antibacterial and antibiofilm properties against MDR-*P. aeruginosa*. When rutin was applied at a low concentration (0.5%), its antibacterial, antibiofilm, and wound healing activities were limited. However, at a higher concentration (1%), rutin supported wound healing activity. Quercetin exhibited remarkable antibacterial, antibiofilm, and wound healing activity compared to rutin at both concentrations (0.5% and 1%). Both polyphenols enhanced the effectiveness of gentamicin in controlling the infection and promoting wound healing when used as an additive in the preparation. Additionally, examining the combined effects of these polyphenols with other antibiotics in controlling polymicrobial biofilms may offer valuable insights for managing infections and diabetic wounds. Moreover, novel formulations of quercetin may help in the improvement of drug delivery, leading to enhanced effects.

## Figures and Tables

**Table 1:** In vitro antibacterial activities of rutin and quercetin against MDR-*P. aeruginosa* (ATCC 27853).

	Antibacterial Activity		
	MIC (µg/mL)	MBC (µg/mL)	FIC Index
Rutin	512	1024	1.5003 *
Quercetin	256	512	1.5017 *
Gentamicin	2	4	

* Indifferent, no interaction.

**Table 2:** Physicochemical properties of ointment.

Parameter	Rutin	Quercetin
Color	Slightly yellowish	Dark yellowish
Odor	Odorless	Odorless
Taste	Bitter	Bitter
Spreadability (s)	13 s	10 s
Diffusion	0.6 cm	0.8 cm
Stability	Stable at 40 °C, 24 °C and 37 °C	Stable at 40 °C, 24 °C and 37 °C
Washability	Satisfactory	Satisfactory
Homogeneity	Good	Good

**Table 3:** Bacterial load in the regenerated skin tissue after different treatments.

Treatment	Log_10_ CFU/g of Tissue
Base	5.981 ± 0.529
Gentamicin (0.1%)	1.254 ± 0.247 ***
Rutin (0.5%)	5.987 ± 0.697
Rutin (1%)	4.987 ± 0.784 *
Quercetin (0.5%)	4.254 ± 0.248 *
Quercetin (1%)	2.954 ± 0.541 ***
Rutin (1%) + Gentamicin (0.1%)	0.885 ± 0.028
Quercetin (1%) + Gentamicin (0.1%)	0.623 ± 0.035 ^+^

All values are mean ± SEM, *n* = 6, * *p* < 0.05, *** *p* < 0.001 indicate significance compared to the base-treated control. ^+^ *p* < 0.05 indicates significance compared to the individual quercetin (1%) treatment.

## Data Availability

The raw data supporting the conclusions of this article will be made available by the authors on request.
