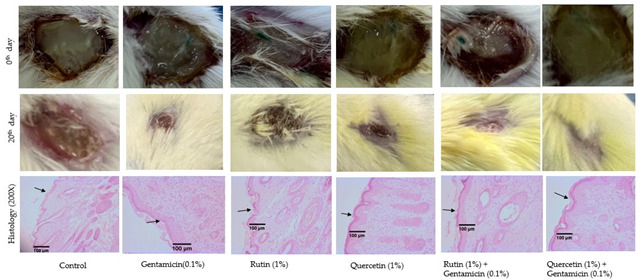# Correction: Almuhanna et al. Antibacterial, Antibiofilm, and Wound Healing Activities of Rutin and Quercetin and Their Interaction with Gentamicin on Excision Wounds in Diabetic Mice. *Biology* 2024, *13*, 676

**DOI:** 10.3390/biology14060617

**Published:** 2025-05-27

**Authors:** Yasir Almuhanna, Abdulrahman Alshalani, Hamood AlSudais, Fuad Alanazi, Mohammed Alissa, Mohammed Asad, Babu Joseph

**Affiliations:** 1 Department of Clinical Laboratory Sciences, College of Applied Medical Sciences, Shaqra University, Shaqra 11961, Saudi Arabia; masad@su.edu.sa; 2 Chair of Medical and Molecular Genetics Research, Department of Clinical Laboratory Sciences, College of Applied Medical Sciences, King Saud University, Riyadh 12372, Saudi Arabia; aalshalani@ksu.edu.sa (A.A.); halsudais@ksu.edu.sa (H.A.); 3 Department of Clinical Laboratory Sciences, College of Applied Medical Sciences, King Saud University, Riyadh 12372, Saudi Arabia; foalanazi@ksu.edu.sa; 4 Department of Medical Laboratory, College of Applied Medical Sciences, Prince Sattam bin Abdulaziz University, Al-Kharj 11942, Saudi Arabia; m.alissa@psau.edu.sa

## Error in Figure 4

In the original publication [1], there was an error in Figure 4. The histology (200×) second image—Gentamicin (0.1%)—did not belong to this study. The figure has been replaced. The scientific conclusions are unaffected. The Academic Editor approved this correction. The original publication has also been updated.